# Validating the inactivation of viral pathogens with a focus on SARS-CoV-2 to safely transfer samples from high-containment laboratories

**DOI:** 10.3389/fcimb.2024.1292467

**Published:** 2024-03-06

**Authors:** Sankar Prasad Chaki, Melissa M. Kahl-McDonagh, Benjamin W. Neuman, Kurt A. Zuelke

**Affiliations:** ^1^ Global Health Research Complex, Division of Research, Texas A&M University, College Station, TX, United States; ^2^ Department of Biological Sciences, Texas A&M University, College Station, TX, United States; ^3^ Department of Molecular Pathogenesis and Immunology, Texas A&M University, College Station, TX, United States

**Keywords:** biosecurity, high-containment, SARS-CoV-2, pathogen inactivation, validation, sample transportation

## Abstract

**Introduction:**

Pathogen leak from a high-containment laboratory seriously threatens human safety, animal welfare, and environmental security. Transportation of pathogens from a higher (BSL4 or BSL3) to a lower (BSL2) containment laboratory for downstream experimentation requires complete pathogen inactivation. Validation of pathogen inactivation is necessary to ensure safety during transportation. This study established a validation strategy for virus inactivation.

**Methods:**

SARS-CoV-2 wild type, delta, and omicron variants underwent heat treatment at 95°C for 10 minutes using either a hot water bath or a thermocycler. To validate the inactivation process, heat-treated viruses, and untreated control samples were incubated with A549-hACE2 and Vero E6-TMPRSS2-T2A-ACE2 cells. The cells were monitored for up to 72 hours for any cytopathic effects, visually and under a microscope, and for virus genome replication via RT-qPCR. The quality of post-treated samples was assessed for suitability in downstream molecular testing applications.

**Results:**

Heat treatment at 95°C for 10 minutes effectively inactivated SARS-CoV-2 variants. The absence of cytopathic effects, coupled with the inability of virus genome replication, validated the efficacy of the inactivation process. Furthermore, the heat-treated samples proved to be qualified for COVID-19 antigen testing, RT-qPCR, and whole-genome sequencing.

**Discussion:**

By ensuring the safety of sample transportation for downstream experimentation, this validation approach enhances biosecurity measures. Considerations for potential limitations, comparisons with existing inactivation methods, and broader implications of the findings are discussed.

## 1 Introduction

When working with highly virulent organisms in high containment laboratories such as Biosafety Levels 3 or 4, the validation of pathogen inactivation methods takes on a heightened level of importance due to the potential risks involved. Several incidents have occurred in the past involving incomplete inactivation of pathogens in high-containment laboratories (H.C, 2008; GAO-16-642, 2016).

Release of live pathogens from high containment laboratories may result in infection and fatality in humans or animals while potentially leading to facility shutdown, restriction in government funding, monetary penalty, and possible jail time. Several methods for viral inactivation have been published in the literature, including but not limited to treating samples with bleach, alcohol, paraformaldehyde, detergent (sodium dodecyl sulfate, Triton X-100, Tween 20, NP-40), Trizol reagent, UV irradiation, and heat (Patterson et al., 2020). Despite variations in the effectiveness of pathogen inactivation by different methods, a common and reliable validation method is warranted to ensure the complete loss of pathogen replicability and infectivity before removal from a high-containment laboratory and transfer to a lower containment laboratory.

SARS-CoV-2, the causative agent of the COVID-19 pandemic, resulted in 1.15 million deaths in the United States (CDC, 2023) and 6.98 million deaths globally (WHO, 2023) as of November 2023. Laboratory researchers throughout the globe are continuously working on vaccine modifications and therapeutic updates to deal with the frequent viral mutations and new SARS-CoV-2 evolution (Markov et al., 2023). With increased SARS-CoV-2 research and clinical diagnosis, the global focus on laboratory biosafety and biosecurity has elevated (Joseph et al., 2022; Rutjes et al., 2023). Transportation of SARS-CoV-2 positive patient samples or live virus culture falls under the packaging and shipping criteria of UN3373 biological substance of category B (DoT, 2020). Manipulation of the SARS-CoV-2 live virus in cell cultures, human samples, or animals requires Biosafety Level 3 (BSL3) or Animal Biosafety Level 3 (ABSL3) containment facilities as recommended by the World Health Organization and the U.S. Centers for Disease Control and Prevention (CDC, 2021; WHO, 2021). A lack of BSL3 laboratories and limited instrumental access makes it difficult to efficiently and timely conduct the research and diagnostic process, necessitating sample transportation to a lower containment laboratory (BSL2). With this in consideration, complete viral inactivation is required before transferring samples to a lower containment laboratory as per biosafety and biosecurity protocol.

Some studies reported the inactivation of SARS-CoV-2 by heat treatment using temperatures ranging from 56°C to 95°C (Kim et al., 2020; Pastorino et al., 2020; Auerswald et al., 2021; Batejat et al., 2021; Biryukov et al., 2021; Gamble et al., 2021; Xiling et al., 2021; Delpuech et al., 2022; Nims & Plavsic, 2022). The stringency of sample inactivation may be limited due to the need for the preserved characteristics of the post-treated sample required for specific downstream applications. However, we opted for a stricter method (95°C for 10 min) of sample inactivation to emphasize post-inactivation validation criteria for high-containment laboratories. Concurrently, the quality of post-treated samples was tested for downstream molecular applications. Unlike the bacterial ability to grow and form colonies on agar plates, viral growth requires live cell support. Plaque assay and 50% cell culture infectious dose (TCID50) assays, typically rely on visible cytopathic effects (CPE), Cell survival is possible upon cytopathic viral infection (Heaton, 2017). Either assay would need multiple blinded personnel to reduce inconsistencies and counting errors (Smither et al., 2013), questioning their independent use in high containment laboratories to validate virus inactivation. A qPCR is not considered a direct measure of viral infectivity as the presence of viral genetic material does not indicate actively infectious particles (Mendoza et al., 2020). Comparing the genome copy number of untreated vs. heat-treated viruses collected at the beginning and end of the cellular infections (24h, 48h, or 72h) can indicate whether the virus is infectious or not, since increases in the genome copy number require cellular infection and replication of the virus. In the case of non-cytopathic viral strains, CPE may not be apparent, making it challenging to assess infectivity through traditional methods like observing cellular damage, though immunostaining can be used to identify foci of infection for non-cytopathic viruses. Using qPCR allows researchers to indirectly assess infectivity by monitoring changes in viral genome copy numbers. This study validated the inactivation of SARS-CoV-2 followed by a quality check of inactivated samples.

## 2 Materials and methods

### 2.1 Virus and cell line

The following reagents were obtained through Biodefense and Emerging Infections (BEI) Resources, NIAID, NIH: (i) Human Lung Carcinoma Cells (A549) Expressing Human Angiotensin-Converting Enzyme 2 (HA-FLAG), NR-53522, (ii) Cercopithecus aethiops Kidney Epithelial Cells Expressing Transmembrane Protease, Serine 2 and Human Angiotensin-Converting Enzyme 2 (Vero E6-TMPRSS2-T2A-ACE2), NR-54970, (iii) SARS-Related Coronavirus 2, Isolate hCoV-19/USA/MD-HP05647/2021 (Lineage B.1.617.2; Delta variant), NR-55672, contributed by Dr. Andrew S. Pekosz, (iv) SARS-Related Coronavirus 2, Isolate hCoV-19/USA-WA1/2020, NR-52281, (v) SARS-Related Coronavirus 2, Isolate hCoV-19/USA/COCDPHE-2102544747/2021 (Lineage B.1.1.529, BA.2; Omicron Variant), NR-56520, and (vi) SARS-Related Coronavirus 2, Isolate USA-WA1/2020, Heat Inactivated, NR-52286. Items (iv), deposited by the Centers for Disease Control and Prevention.

### 2.2 Cell culture

A549-hACE2 cells and Vero E6-TMPRSS2-T2A-ACE2 cells were cultured in Dulbecco’s Modified Eagle’s Medium with high glucose (Thermo Fisher Scientific # 11965092), supplemented with 10% fetal bovine serum and 1% Antibiotic-Antimycotic (Thermo Fisher Scientific # 15240062). The cells were incubated in a 37°C incubator with 5% CO2 and >90% humidity. For sub-culture, the cell culture media was aseptically removed, and the cell layer was rinsed twice with Ca2+- and Mg2+-free Dulbecco’s phosphate-buffered saline (DPBS) to eliminate all traces of serum. Subsequently, 2 to 3 mL of 0.25% trypsin-EDTA was added to the culture flask, and the flask was incubated at 37°C until the cell layer dispersed (typically within 5 minutes) without agitating the cells to prevent clumping. Once dispersed, additional media (10-20 ml) was added to neutralize trypsin, and the mixture was centrifuged at 200 g for 5 min to discard the supernatant. Cells were resuspended in fresh culture media for counting and plating. Generally, cells from one confluent flask were distributed to approximately 8 similar flasks for subculture and plating.

### 2.3 Virus inactivation

Frozen viral stocks and active cell culture supernatants are two widely used types of virus samples in virology research laboratories, and both were used in this study. Virus heat inactivation procedures were conducted by subjecting samples to a temperature of 95°C for 10 minutes using two commonly used laboratory instruments: (i) a hot water bath and (ii) a thermocycler. A hot water bath is cost-effective and versatile (sample volume and container types) but may have challenges in temperature control. In this investigation, 1 ml of rapidly thawed frozen virus stock in a securely tighten screw-capped cryovial underwent heat inactivation in a pre-calibrated hot water bath set at 95°C with a securely closed lid. A thermocycler offers precise control and uniform heating but comes with a higher cost and need of specific container like PCR tubes or plates. In this study, we concurrently heat-inactivated 96 virus samples using a sealed 96-well plate, with each well accommodating a volume of 0.050-0.2 ml. The thermocycler was set with a block temperature of 95°C and a lid temperature of 105°C.

### 2.4 Quantitation of genome copy numbers by RT-qPCR

RT-qPCR was performed to quantify genome copy numbers before and after heat inactivation as per the earlier protocol (Chaki et al., 2022). In brief, 0.05 ml of sample was diluted in 0.05 ml lysis buffer (2% TBE, 1% Tween 20) and heat-lysed at 95°C for 15 min. Following heat lysis, a 200-fold diluted sample in sterile water was used directly in PCR. RT-qPCR was performed using the CDC N1-F: 5’GACCCCAAAATCAGCGAAAT 3’, CDC N1-R: 5’ TCTGGTTACTGCCAGTTGAATCTG 3’, Probe CDC N1: 5’ FAM-ACCCCGCATTACGTTTGGTGGACC-BHQ1 3’ and Luna Universal Probe One-Step RT-qPCR Kit (Catalog No. E3006, NEB, Ipswich, MA, US) in a BIORAD CFX96 thermocycler. Viral genome copies were quantified against a standard curve generated using heat-inactivated SARS-CoV-2 (BEI resource, NR-52286). Alternatively, direct CT value comparison may also be used to validate inactivation.

In a typical experimental procedure, samples were thoroughly mixed with lysis buffer in 96-well plates. The plates were sealed with adhesive foil and lysed for 10 minutes in a thermocycler at 95°C. Notably, we opted not to purify RNA for RT-qPCR, instead using the heat-lysed samples directly. This approach aimed at simplifying the process, reducing costs, and facilitating a rapid high-throughput assay setup in a BSL3 facility. Sample dilution was performed with sterile water to ensure no interference of phenol red-containing media in qPCR fluorescent detection. A fixed sample volume of 0.007 ml was used for serial sample dilution and easy transfer to the PCR plate using a 12-channel micropipette. Within a biosafety cabinet, plates were organized sequentially as one 96-well plate of lysed sample, two plates pre-filled with 0.063 ml of sterile water in each well, and one plate pre-filled with 0.013 ml of RT-qPCR reagent mixtures in each well. Plates were sealed, spun down, and placed in the thermocycler. The PCR cycle steps included incubation at 55°C for 10 min (1 cycle), 95°C for 1 min (1 cycle), and 41 cycles of 95°C for 10 s and 60°C for 30 s. The entire PCR process took approximately 1 hour and 8 minutes. Data generated were processed using Bio-Rad-CFX Maestro and analyzed using Microsoft Excel and GraphPad Prism 9 software.

### 2.5 Analyzing cytopathic effect

At the end of viral incubation (72 h), samples were collected for RT-qPCR and the remaining media was aspirated. Plates were fixed in 10% buffered formalin for 30 min and stained with 1% crystal violet for 1-5 min. Following gentle washing in water, plates were dried in room temperature. Dry plates were inspected visually as well as scanned under a document scanner. Microscopic observation and image collection were performed using a brightfield microscope to examine any cytopathic effects. Untreated cells started at 80% confluency are expected to cover the whole cell culture surface area by 72 h of incubation. Thus, any unstained empty spot against blue stained cell background is indication of presence of live virus or inactivation failure. While absence of any empty white spot against the blue stained cell background that covers the whole cell culture surface area is considered inactivation success and validate the inactivation process. As there is chance of error in identifying cytopathic effect originating during cell plating, staining, washing and visualization steps, a secondary confirmation of virus inactivation using RT-qPCR is warranted.

### 2.6 The strategy of workflow

**Figure 1:**
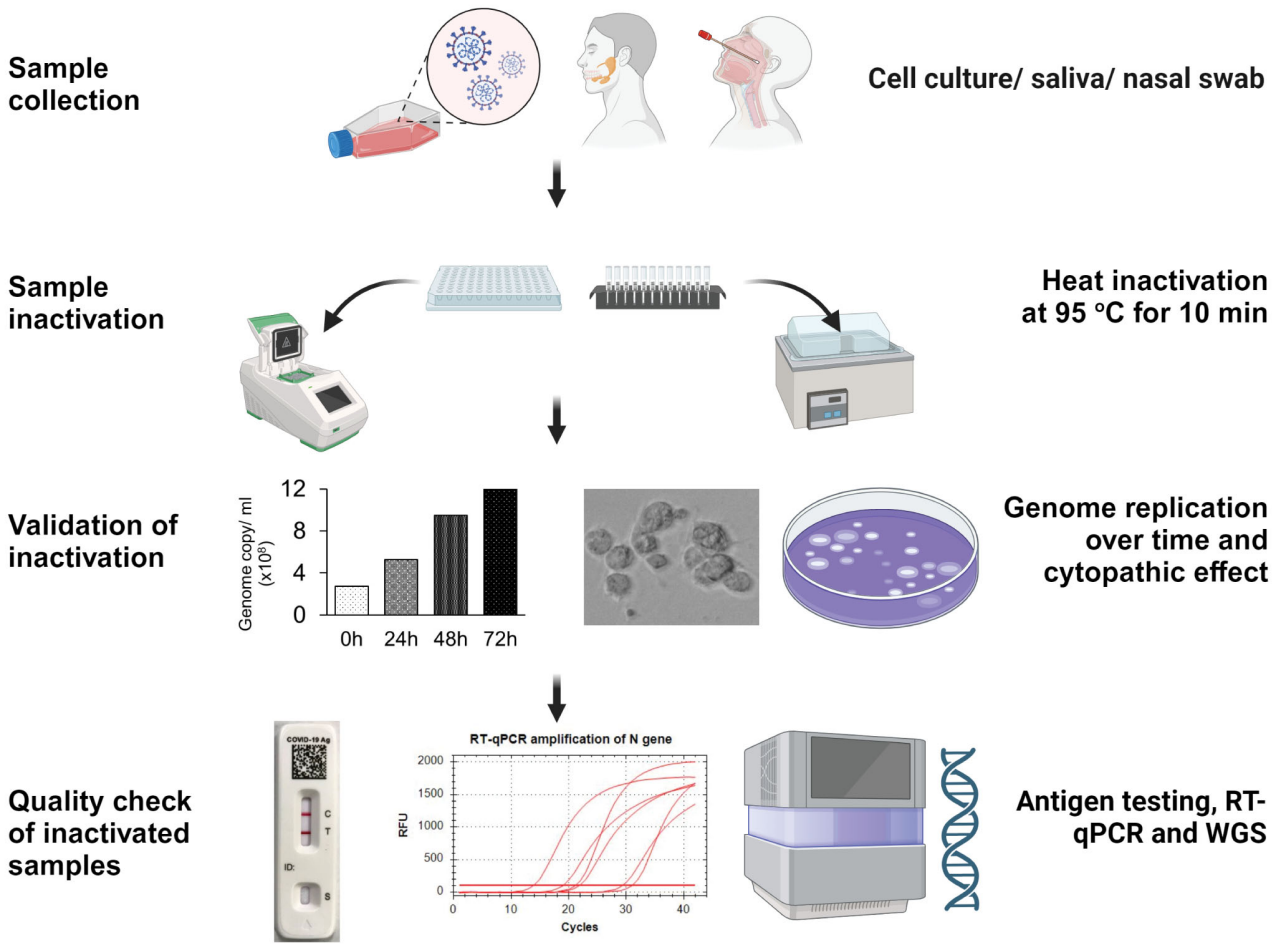
Workflow strategy for validating pathogen inactivation in a high containment laboratory. This figure illustrates the workflow, including sample collection, inactivation, validation, and quality assessment of inactivated samples for downstream applications.

Workflows proceed from sample collection to heat inactivation, validation of sample inactivation, and post-inactivation sample quality check. In the inactivation step, SARS-CoV-2 positive samples, including cell culture supernatant, saliva, or nasal swab samples, distributed in a 96-well PCR plate or 2 ml screw-capped tubes, are heat-inactivated at 95°C for 10 min in thermocycler or hot water bath. In the validation step, pre- or post-inactivated viral samples are used to infect A549-hACE2 cells and monitor virus genome replication and the cytopathic effect over time. Finally, the quality of inactivated samples is checked by COVID-19 antigen testing, RT-qPCR of N-gene amplification, and whole genome sequencing (**Figure 1**).

### 2.7 Validation following virus inactivation using hot water bath

SARS-CoV-2 Delta variant (NR-55672) was initially propagated in A549-hACE2 cells and stored frozen as 1 ml aliquot (5.9x10^9^ genome copies or 2.4x10^7^ TCID50/ml) in 2 ml screw-caped cryovials. For inactivation, rapidly thawed frozen virus stock was heat-treated in a 95°C hot water bath for 10 minutes. For validation of heat inactivation, pre-treated (untreated) and post-treated samples were serially diluted 10-fold (1/10 to 1/1000000) in cell culture media. Subsequently, 0.1 ml of diluted virus/well (N=3) was used to infect 80% confluent monolayers of A549-hACE2 cells in 0.2 ml of DMEM culture media (total volume of 0.3 ml) in a 48-well cell culture plate. Half of the plate was incubated with the heat-treated virus and the rest with the untreated control virus for 72 h. Cells were incubated at 37°C incubator with 5% CO_2_ and at >90% humidity. Cell culture supernatant was collected at 0 h, 24 h, 48 h, and 72 h time points to analyze genome replication by RT-qPCR. At the end of 72 h incubation, cells were fixed and stained with crystal violet to examine the cytopathic effects on permissive cells.

### 2.8 Validation following virus inactivation using thermocycler

In the context of a commonly used antiviral drug study, 80% of a confluent monolayer of A549-hACE2 cells in two 48-well cell culture plates were initially infected with the SARS-CoV-2 Delta variant (MOI 0.16, frozen stock) to cultivate a fresh and actively replicating working virus stock. Following 72 hours of incubation, cell culture supernatants (0.01 ml from each well) from the two 48-well plates were combined into a single 96-well plate (n=96 samples) in duplicates (replica plate). Following the determination of the genome copy number of each sample through RT-qPCR, one plate underwent heat treatment in a thermocycler, while the other served as a pre-treated control. For validation, we infected 80% confluent monolayers of A549-hACE2 cells in 96-well plates with the untreated or heat-treated virus. Cells were incubated at 37°C incubator with 5% CO2 and at >90% humidity. After 72 h of incubation, cell culture supernatants were collected to analyze genome replication by RT-qPCR. At the end of 72 h incubation, cells were fixed and stained with crystal violet to examine the cytopathic effects on permissive cells.

### 2.9 Validation of inactivation of various types of SARS-CoV-2

In this experiment, we used BEI Resources of SARS-CoV-2 isolate USA-WA1/2020 (NR-52281) as wild type (WT), Delta variant (NR-55672), and Omicron BA.2 variant (NR-56520). We isolated the Omicron BQ.1.1 variant from a nasal swab sample using Vero E6-TMPRSS2-T2A-ACE2 cells. Viruses in 1 ml aliquot in screw-capped cryovials were subjected to heat treatment in a hot water bath at 95°C for 10 minutes. To validate the effectiveness of this heat inactivation, 80% confluent monolayer of A549-hACE2 cells or Vero E6-TMPRSS2-T2A-ACE2 cells in 24-well or 96-well cell culture plates were infected with treated or untreated virus (MOI of 0.16) for one hour only. After washing with DPBS, cells were incubated in fresh DMEM media. We did a two-step validation of inactivated samples by blind-passaging for 72 hours, then transferring supernatant onto fresh permissive cells and incubating again for 72 hours. Cell culture supernatant was collected at 0 h, 24 h, 48 h, and 72 h post-infection for RT-qPCR. After 72 hours of incubation, cells were fixed and stained with crystal violet to examine the cytopathic effects.

### 2.10 Validation of virus inactivation using fluorescent reporter-based assay

For validation of heat-inactivation, the SARS-CoV-2 expressing Venus fluorescent reporter gene (Morales Vasquez et al., 2022) was subjected to heat treatment in a screw-capped cryovial at 95°C water bath for 10 minutes. To validate inactivation, 80% Confluent monolayers of A549-ACE2 or Vero E6-TMPRSS2-T2A-ACE2 cells, grown in a black 24-well clear-bottom plate (ibidi # 82426), were incubated with treated or untreated virus (MOI of 0.16) for only one hour at 37°C, 5% CO_2_. After one hour of infection, cells were washed in PBS and incubated in Phenol-red-free complete DMEM media for various time durations at 37°C, 5% CO_2_. Cell culture supernatant was collected at 0 h, 24 h, 48 h, and 72 h post-infection for RT-qPCR. After 72-hour of incubation, cells were fixed in 4% PFA for 20 min and permeabilized in 0.3% Triton X-100 in PBS for 5 min. Cell nuclei were stained with NucBlue (Hoechst 33342, Thermo Fisher Scientific) reagent for one hour and replaced with fresh PBS for imaging under a Zeiss Axio Vert. A1 FL-LED microscope equipped with Plan-Apochromatic objectives and Axiocam 305 mono camera. Images were processed and analyzed using Fiji. Green-fluorescence intensity of were quantified from randomly selected 24 images from treated or untreated group.

### 2.11 Heat-inactivated sample quality check for COVID-19 antigen testing

Although the establishment of validation criteria was the major emphasis of this article, we also checked the quality of post-inactivated samples for downstream molecular applications. Lateral‐flow immunoassay-based SARS-CoV-2 antigen tests are a quick alternative to the RT‐PCR method. We used the Flowflex COVID-19 Antigen Home Test kit (ACON Laboratories, Inc., Sandiego, CA, USA) to check the inactivated sample quality. Pre- or post-treated samples (SARS-CoV-2 culture, human saliva or nasal swabs) were diluted 1:1 in the kit-provided sample dilution buffer and tested as per the manufacturer protocol. We repeated the experiments two times.

### 2.12 Heat-inactivated sample quality check for the application of virus detection by RT-qPCR

To test the quality of heat-inactivated samples for virus detection, N-gene-targeted RT-qPCR was carried out using the CDC N1 oligo pair and FAM probe as per the protocol described above. In brief, 0.007 ml of 1:1 diluted sample in lysis buffer, before or after heat treatment (95°C for 10 min), were used in a 0.020 ml RT-qPCR reaction to amplify the SARS-CoV-2 N gene segment.

### 2.13 RNA extraction and whole genome sequencing of SARS-CoV-2 obtained from heat-inactivated saliva samples

To test the genome quality of SARS-CoV-2 in heat-inactivated samples, whole genome sequencing was performed. Viral RNA was isolated from pre-screened heat-inactivated positive saliva samples using the Monarch Total RNA Miniprep Kit (NEB, Cat # T2010S, Ipswich, MA, United States) with DNase treatment according to the manufacturer’s instruction. cDNA was synthesized from 6 ml of RNA using SuperScript™ IV First-Strand Synthesis System (Invitrogen, Cat. 18091050, CA, USA). The resulting cDNA served as the template for subsequent reactions. Library preparation was performed using the Swift SNAP SARS-CoV-2 amplicon panel, followed by sequencing using Illumina NextSeq 550. Approximately 1 million reads per sample were obtained, mapped, and assembled using BWA. Sequences were deposited to GISAID for open access.

### 2.14 Statistical analysis

Each experiment was repeated two to three times independently. Box plot, Bar diagrams and line charts were generated using GraphPad Prism 9.5.1. and data were expressed as Mean ± SD. T test and analysis of variance (ANOVA) were performed using GraphPad Prism 9.5.1. Data were considered significant at P ≤ 0.05.

## 3 Results

### 3.1 Validation of virus inactivation following treatment in a hot water bath

Heat treatment of the SARS-Related Coronavirus 2, Isolate hCoV-19/USA/MD-HP05647/2021 (Lineage B.1.617.2; Delta variant), NR-55672 frozen subculture in a 95 °C hot water bath for 10 minutes resulted in the complete inactivation of the viruses, rendering them noninfectious and incapable of replication. To validate the efficacy of this heat inactivation, A549-hACE2 cells were exposed to the heat-treated viruses (n=3 samples/group) over varying time intervals to monitor genome replication and the presence of any cytopathic effects.

**Figure 2:**
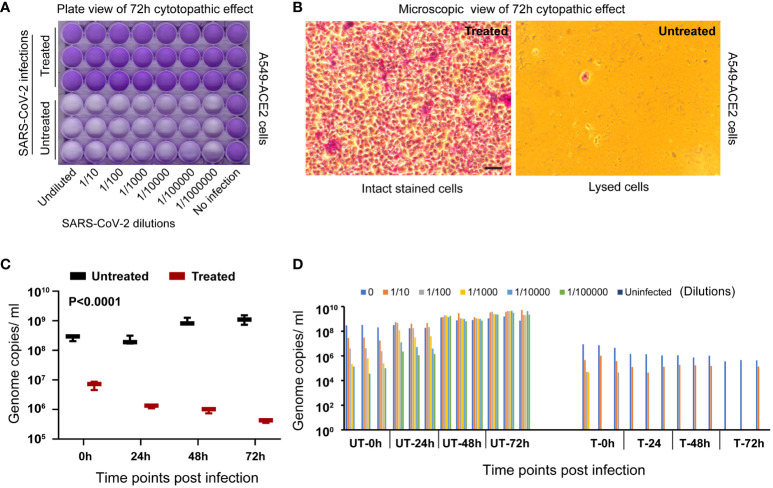
Validation of virus inactivation following treatment in a hot water bath. **(A)** The entire plate scan of A549-ACE2 cells, incubated with either untreated or heat-treated SARS-CoV-2 Delta variant, reveals intact stained cells covering the entire cell surface area in the treated group. This contrasts with the unstained lysed cell areas observed in the untreated group, validating the inactivation process in the treated group. **(B)** A representative microscopic view of cells from each group is presented as observed under a 20x brightfield objective (Scale bar indicating 50 micrometers). **(C)** RT-qPCR targeting the virus spike protein N1 gene segment demonstrates a significant decrease (P<0.0001) in genome replication in the treated group compared to the untreated group at 72 hours post-incubation and further validates the inactivation process. **(D)** The bar diagram illustrates individual samples’ genome replication status (n=3 per dilution group per time point) at various dilutions and time points post-incubation.

Visual inspection under a brightfield microscope and endpoint imaging employing crystal violet staining demonstrated a notable absence of any cytopathic effect. The cells incubated with the heat-treated viruses remained healthy and intact throughout the observation period, providing compelling evidence for the effectiveness of heat inactivation. In contrast, A549-hACE2 cells exposed to untreated virus specimens displayed clear signs of infection and subsequent cell lysis, culminating in unstained empty wells (**Supplementary Figure S1**; **Figures 2A, B**). This finding serves as one type of validation of virus inactivation.

Recognizing that the early detection of cytopathic effects can be challenging and potentially subject to observer or cell type bias, we employed RT-qPCR to quantitatively evaluate the viral genome replication at various time points post-incubation with A549-hACE2 cells. In the group exposed to heat-treated viruses, there was a progressive decrease in viral genome copy numbers over time, representing the natural decay of inactive viral genetic material (two-way ANOVA, P<0.0003). Conversely, the group exposed to untreated viruses exhibited a gradual increase in viral genome copy numbers over time (Two-way ANOVA, P<0.0042), indicative of successful inactivation in the treated group in contrast to the ongoing viral replication within the untreated group (**Figure 2C**). A significant (P<0.0001, n=3/group) disparity in viral genome copy numbers was observed between the heat-treated and untreated groups at different time points as analyzed using ANOVA. The validation process was further reinforced by employing a dilution series of the virus. Lower viral loads resulting from higher sample dilutions resulted in lower genome copies, as expected (**Figure 2D**). This finding serves as a dual simultaneous validation of heat inactivation.

### 3.2 Validation of virus inactivation following heat treatment in a thermocycler

**Figure 3:**
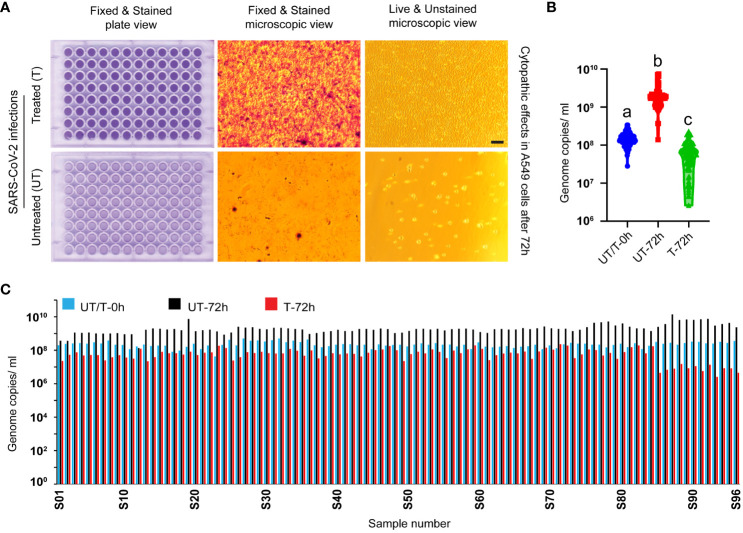
Validation of virus inactivation following heat treatment in a thermocycler. **(A)** Figure represents images of A549-ACE2 cells incubated with either heat-treated (top panel) or untreated (bottom panel) SARS-CoV-2 Delta variant. Whole-plate scans and microscopic image views reveal intact cells covering the entire cell surface area in the treated group, contrasting with the lysed empty cells in the untreated group, validating the inactivation process (scale bar 50 micrometers). **(B)** RT-qPCR targeting the virus spike protein N1 gene segment demonstrates a significant decrease in genome replication in the treated group at 72 hours post-incubation (P<0.0001 among a, b, and c in one-way ANOVA). This robust finding further validates the inactivation process. **(C)** The bar diagram illustrates the genome replication status of individual samples (n=96 per group) at various time points post-incubation. UT/T-0h indicates the viral loads that were distributed equally to the untreated and treated groups before heat treatment.

Heat treatment of SARS-CoV-2 Delta variant (NR-55672) fresh cell culture supernatant in a 96-well plate in a thermocycler effectively inactivated the virus and rendered it non-infectious. This inactivation was corroborated by the absence of any cytopathic effect in A549-hACE2 cells, which were visually inspected and imaged. Cells incubated with the treated virus (n=96) remained healthy, stained, and covered the entire surface area 72 hours post-incubation (**Figure 3A**, top panel), thus validating the successful inactivation of the virus. Conversely, cells incubated with the untreated virus samples (n=96) exhibited complete lysis, leaving behind unstained empty wells (**Figure 3A**, bottom panel).

To further validate the inactivation of the virus, genome replication was compared through RT-qPCR at two time points: 0 hours and 72 hours post-incubation with A549-hACE2 cells. In the heat-treated group, there was either a decrease in viral genome copies number or no significant change, confirming the failure of viral replication and the inactivation of the virus. In contrast, the untreated virus displayed an increase in genome copy numbers 72 hours post-incubation with A549-hACE2 cells (**Figures 3B, C**). One way ANOVA showed significant difference among the different groups (P<0.0001, n=96 in each group). This comprehensive analysis clearly demonstrates the validation of the heat treatment in inactivating the SARS-CoV-2 Delta variant.

### 3.3 Validation of inactivation of various types of SARS-CoV-2

**Figure 4:**
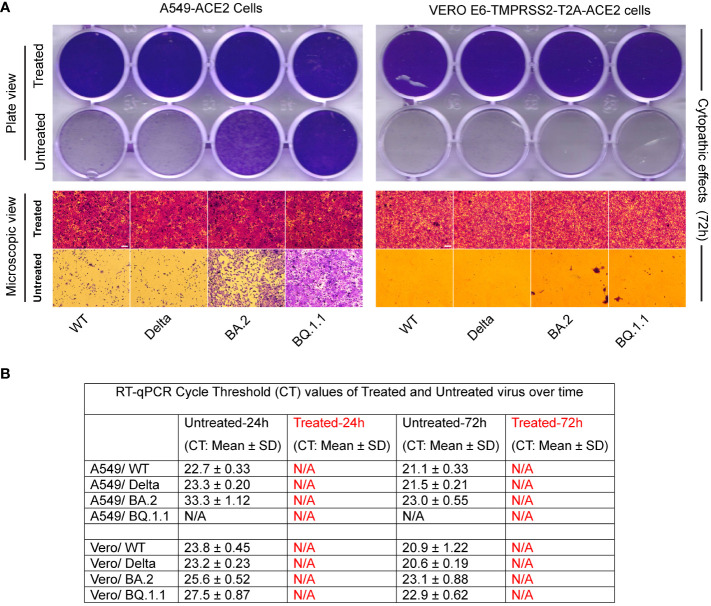
Validation of various types of SARS-CoV-2 inactivation. **(A)** Figure represents images of A549-ACE2, and Vero E6-TMPRSS2-T2A-ACE2 cells incubated with either heat-treated or untreated SARS-CoV-2 variants. Whole-plate scans (top panel) and microscopic image views (bottom panel) reveal intact cells covering the entire cell surface area in the treated group, contrasting with the lysed or fewer cells in the untreated group, validating the inactivation process (scale bar 50 micrometers). **(B)** RT-qPCR of N1 gene replication revealed absence of Cycle Threshold (CT) values (N/A) in the treated groups compared to a decrease in CT values with incubation (indicating an increase in genome copy number) in the untreated group **(B)**.

When different variants of SARS-CoV-2 (Wild type, Delta, Omicron BA.2, and Omicron BQ.1.1) were subjected to heat treatment in a hot water bath at 95°C for 10 minutes, they all underwent complete inactivation. Visual inspection and endpoint imaging using crystal violet staining revealed the absence of any cytopathic effect in the cell cultures exposed to heat-treated viruses. These cells remained healthy and intact, covering the whole surface area of the wells, thus validating the efficacy of heat-inactivation (**Figure 4A**). In contrast, when cells were similarly exposed to the untreated virus, clear indications of infection and subsequent cell lysis were observed over time. This was evidenced by the presence of unstained empty wells in the cell cultures (**Figure 4A**) by the 72-hour post-infection mark. Interestingly, A549-ACE2 cells were less permissive than VERO E6 TMPRSS2-ACE2 cells to SARS-CoV-2 Omicron variants (BA.2 and BQ.1.1) as reflected in the cytopathic effect and RT-qPCR CT values (**Figure 4**).

RT-qPCR analysis was conducted to compare CT values at different time points to provide an assessment of genome replication. The heat-treated virus group exhibited the absence of CT values (designated as N/A), indicating the lack of cellular infection during the initial hour of infection, leading to a lack of ability of genome replication in this group even after repassage. In contrast, the untreated virus group displayed a decrease in the CT values over time (except BQ.1.1 in A549-ACE2 cells), which signifies an increase in genome copy numbers (**Figure 4B**).

### 3.4 Validation of virus inactivation using fluorescent reporter-based assay

**Figure 5:**
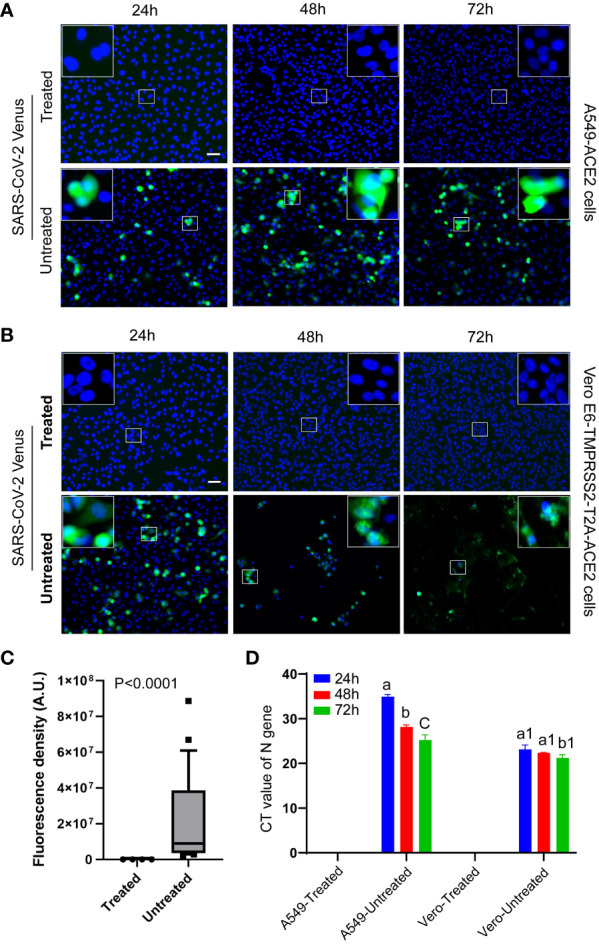
Validation of virus inactivation using fluorescent reporter-based assay. Representative fluorescent images of cells displayed the absence of SARS-CoV-2-Venus (green) expression in treated groups (**A, B**, top panels) and the presence of SARS-CoV-2-Venus (green) in untreated groups (**A, B**, bottom panels), confirming heat inactivation. Magnified square insets in the upper image corner correspond with the central small square area. **(C)** Quantitative image analysis (n = 24/group) shows the absence of virus-specific green fluorescence signal in the treated group and a significant increase (P<0.0001, t test) in the green fluorescence signals in the untreated groups. **(D)** The Cycle Threshold (CT) value of RT-qPCR shows a lack of viral infection/genome replication over time in the heat-treated virus group in contrast to a significant decrease (P<0.0001; a vs. b/c, P<0.05; a1 vs. b1, ANOVA) in CT values (viral infection and genome replication) over time in the untreated virus group. Different letters indicate significant differences within the groups. The scale bar represents 50 micrometers.

Heat treatment of SARS-CoV-2, expressing Venus fluorescent reporter gene, in a 95°C water bath completely inactivated the virus, rendering it noninfectious and incapable of replication. No virus-specific fluorescence signal was detected in the cells incubated with the heat-treated virus at any time point, validating that the heat treatment had inactivated the virus (**Figures 5A, B**, top panels, and **Figure 5C**). In contrast, cells incubated with the untreated virus showed significant increase (P<0.001, t test) in virus-specific fluorescence signals (green), indicating active virus replication (**Figures 5A, B**, bottom panels, and **Figure 5C**). The Vero E6-TMPRSS2-T2A-ACE2 cells were found to be more permissive to the virus and exhibited early cytopathic effect. This was evident in higher fluorescence signals at 24 hours with cytopathic effect and fewer fluorescence signals by 48 and 72 hours which is attributed to excessive cell lysis (**Figure 5C** bottom panel).

To further validate the inactivation of the virus, RT-qPCR was performed to measure Cycle Threshold (CT) values. The heat-treated virus group showed a lack of CT values, indicating failure of genome replication, even after subsequent repassages, validating that the virus was inactivated. In contrast, the untreated virus group displayed a decrease in CT values, indicating an increase in genome copy number, suggesting virus replication in these cells (**Figure 5D**).

### 3.5 Heat-inactivated SARS-CoV-2 maintained antigen quality of detection by lateral flow assay

**Figure 6:**
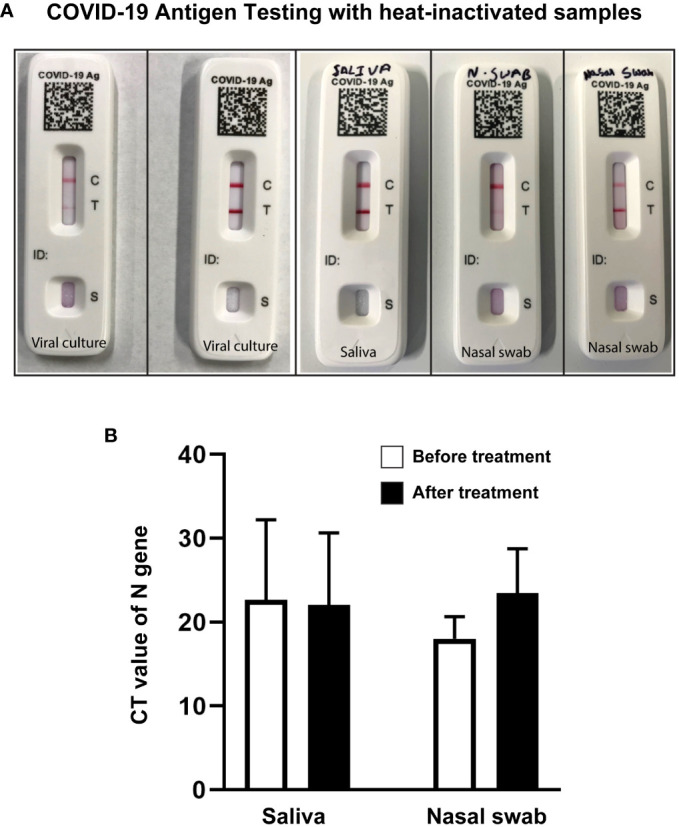
Heat-treated samples qualified for COVID-19 antigen testing and RT-qPCR. **(A)** Representative images of COVID-19 antigen testing (Flowflex, ACON Laboratories, Inc., CA) using heat-treated virus culture or heat-treated known positive samples showing positive test bands. Samples from left to right: low titer viral culture (faint test band), high titer viral culture (dark test band), high titer saliva (dark test band), low titer nasal swab (faint test band), and high titer nasal swab samples (dark test band). **(B)** Bar diagram representing CT value of N Gene Targeted RT-qPCR of positive saliva and nasal swab samples before and after heat treatment. T-test analysis revealed no significant changes in CT values before and after heat treatment.

We performed a lateral-flow immunoassay using an over-the-counter COVID-19 Ag test kit. Pre- and post-heat-treated samples diluted (1:1) in the kit provided solution was used following kit protocol. Heat-treated samples maintained antigen quality and detected positive in the test as expected. Lower viral load in samples resulted in the formation of faint positive test signals as opposed to strong positive test signals obtained from high viral load samples (**Figure 6A**). Samples with high viral load showed a quicker appearance of positive band (T) than low viral load samples. Certain samples yielded inconclusive results, possibly due to low titer, sample degradation during inactivation, or issues with the kit utilized (**Supplementary Figure S2**).

### 3.6 Heat-treated COVID-19-positive saliva and nasal swab samples tested positive in RT-qPCR

To check the effect of heat treatment on sample quality for RT-qPCR, positive saliva and nasal swab samples were tested before and after heat treatment. Samples that tested positive before heat treatment were all positive after heat treatment in RT-qPCR. Despite changes in CT values, the one-way ANOVA analysis indicated no significant (NS; P=0.562) difference between the pre-heat treatment and post-heat treatment conditions (**Figure 6B**).

### 3.7 Whole genome sequencing of SARS-CoV-2 obtained from heat-inactivated saliva samples

SARS-CoV-2 RNA was extracted from heat-treated saliva samples. Following cDNA synthesis and library preparation, genome sequencing was performed using the Illumina sequencing platform. High-quality sequences of >90% SARS-CoV-2 genome coverage were obtained. We have successfully differentiated various lineages of SARS-CoV-2 by whole genome sequencing of viral genome isolated from heat-treated saliva samples (**Table 1**).

**Table 1:** Whole genome sequence analysis of SARS-CoV-2 isolated from heat-inactivated samples.

Specimen	Collection Date	GISAID Accession ID	Pango Lineage	GISAID Clade	Nextstrain Clade	Genome Coverage
Saliva 1	02.05.2021	EPI_ISL_1626969	B.1	G	20A	99.8%
Saliva 2	03.27.2021	EPI_ISL_2365356	B.1	G	20G	100%
Saliva 3	11.04.2020	EPI_ISL_2340581	B.1	GH	20C	98.6%
Saliva 4	01.28.2021	EPI_ISL_2340662	B.1	GH	20A	98.9%
Saliva 5	01.05.2021	EPI_ISL_2340614	B.1.1	GR	20B	99.1%
Saliva 6	01.05.2021	EPI_ISL_2340615	B.1.1	GR	20B	99%
Saliva 7	01.13.2021	EPI_ISL_2340626	B.1.1.519	GR	20B	99.2%
Saliva 8	04.01.2021	EPI_ISL_2340570	B.1.1.7	GRY	20I (Alpha)	100%
Saliva 9	04.06.2021	EPI_ISL_2340575	B.1.1.7	GRY	20I (Alpha)	100%
Saliva 10	01.09.2021	EPI_ISL_2402571	B.1.1.7	GV	20E (EU1)	92.7%
Saliva 11	07.23.2021	EPI_ISL_13295050	P.1.12	GR	20J (Gamma)	99.5%
Saliva 12	01.31.2022	EPI_ISL_10271777	BA.1.1	GRA	21k (Omicron)	99.8%
Nasal Swab	02.21.2023	EPI_ISL_17371329	BQ.1.1	GRA	22E (Omicron)	96.3%

## 4 Discussion

Validating pathogen inactivation is crucial when working with vaccines, therapeutic products, decontamination agents or procedures, clinical samples, or research materials to prevent residual infectivity. High-containment laboratories are often subject to strict regulatory oversight. Before any pathogen inactivation procedure is carried out, a robust and validated inactivation protocol is developed. Validation studies must align with relevant regulatory guidelines and standards, such as those set by the World Health Organization (WHO), the Centers for Disease Control and Prevention (CDC), and the relevant national regulatory agencies (PAHO-WHO, 2014; HHS, 2020). Regulatory agencies, such as the FDA, require extensive data and evidence to demonstrate the effectiveness, safety, and consistency of inactivation methods. Several factors can influence the effectiveness of pathogen inactivation processes, and the choice of inactivation methods is based on downstream applications (Elveborg et al., 2022) where inactivated samples don’t interfere with the assay and provide similar results to pre-inactivated samples. Successful pathogen inactivation requires a comprehensive understanding of the pathogen types (viruses, vegetative bacteria, or more resistant bacterial spores and prions), organic content in samples (cell cultures, body fluids, or other biological materials can protect pathogens from inactivation), sample volume and concentration (higher volume and concentration may necessitate more robust inactivation process), choice of inactivation method (chemical, autoclaving, irradiation), and environmental controls (temperature, humidity, airflow pH levels, contact time). High-containment laboratories often employ chemical treatments, heat, radiation, or a combination of these to inactivate pathogens (Elveborg et al., 2022).

SARS-CoV-2, the virus responsible for COVID-19, is sensitive to heat and can be inactivated at high temperatures. Viruses become less stable and lose infectivity as temperatures increase or as exposure time at a given temperature increases (Biryukov et al., 2021; Herder et al., 2021; Muramoto et al., 2023). This relationship is conveyed through the term D value (decimal reduction value). The D value is the time required in minutes at a given temperature to result in a 1 log10 reduction viral titer. The specific temperatures and durations required for obtaining a desired log10 reduction in titer depend on the virus; for instance, the D value for SARS-CoV-2 has been estimated to be 6 minutes at 56°C (Nims and Plavsic, 2022). The specific temperature and duration required for complete inactivation can vary (Pastorino et al., 2020; Batejat et al., 2021; Delpuech et al., 2022). Higher temperatures and longer exposure times always result in increased inactivation for any given viruses. This study validated that heat treatment of highly transmissible and infectious SARS-CoV-2 and its variants (Delta variant, Omicron BA.2, and Omicron BQ.1.1 variants) at 95°C for 10 minutes inactivated the virus completely. Omicron has more spike protein mutations and exhibits longer survival ability on plastic and human skin surfaces however, the “physical strength” of different SARS-CoV-2 variants in terms of resistance to alcohol treatments remains relatively similar (Hirose et al., 2022). Both inactivation and validation procedures have limitations. The effectiveness of heat treatment can also vary significantly based on the procedure used. Placing samples in open or uncovered containers may substantially decrease the speed and efficiency of virus inactivation through heat treatment (Gamble et al., 2021).

Validating the infection ability of a virus after inactivation involves assessing whether the virus’s ability to infect cells has been compromised or eliminated due to inactivation treatment. This process is crucial for ensuring that inactivation-treated samples are safe and no longer pose a risk of infection. To determine whether a virus is truly inactivated, researchers would subject the treated sample to conditions conducive to virus growth (such as culturing cells susceptible to the virus) and observe whether the virus can replicate and cause infection. This study used A549-ACE2 cells and Vero E6-TMPRSS2-T2A-ACE2 cells to validate virus inactivation. A549-ACE2 cells are derived from human lung tissue (A549 cells), which express the angiotensin-converting enzyme 2 (ACE2) receptor that SARS-CoV-2 uses to enter host cells. Since the primary target of SARS-CoV-2 in the human body is the respiratory system, using ACE2-expressing human lung cells can provide a more physiologically relevant model for studying SARS-CoV-2 viral infection, replication, and host responses in the context of COVID-19. Although Vero E6 cells are frequently used for virus production, in our initial trials, we failed to culture the Omicron variant from saliva or oral swab samples in Vero E6 cells but successfully used Vero E6-TMPRSS2-T2A-ACE2 cells. When a virus infects a host cell, it can cause observable changes in the morphology and behavior of the cell. These changes, collectively known as the cytopathic effect (CPE), can include cell rounding, cell detachment, cell lysis, formation of giant multinucleated giant cells, and other cellular abnormalities (Suchman & Blair, 2007). Virus replication damages the host cell, resulting in cytopathic effects. Commonly used assays developed based on CPE are the plaque assay (PFU/ml), 50% tissue culture infectious dose assay (TCID50/ml), and Specific Infection (SIN/ml) calculation assay (Smither et al., 2013; Mendoza et al., 2020; Cresta et al., 2021). Before and after inactivation, scientists can examine the host cells for the presence or absence of CPE. If the inactivation process is effective, there should be an absence of CPE in treated samples compared to untreated samples, as observed in this study (**Figures 2A, B**, **3A**,**4A**). The absence of CPE after inactivation can provide evidence and validate that the virus is no longer capable of causing infection in host cells. This ensures sample transportation safety to lower containment laboratories for downstream applications such as vaccine production or diagnosis. Monitoring the expression of fluorescent signals over time can also reflect if the virus is inactivated or infectious and replicating inside the cells, which was demonstrated in this study (**Figures 5A–C**). While the untreated positive control exhibits fluorescence due to viral growth after a 24-hour incubation period, extending the incubation to 48 or 72 hours enhances confidence in validating inactivation for treated samples lacking fluorescence signals.

While our validation strategy is applicable to other virus types, the choice of a cell line may vary based on the cytopathic response, as not all cell lines are equally responsive to all virus types. Some cells may be more susceptible to infection and display a cytopathic effect (virus-induced damage to cells), while others may be less susceptible. In this study, Vero E6-TMPRSS2-T2A-ACE2 cells were more permissive to the SARS-CoV-2 Omicron variant compared to ACE2-A549 cells (**Figure 4**). This suggests that the ability of a virus to infect and replicate in different cell types can vary significantly.

While CPE is a valuable tool for assessing virus inactivation, it is not the only method used. Molecular assays, such as qPCR, can be employed with CPE analysis to validate virus inactivation thoroughly (Zhang et al., 2019; Cassedy et al., 2021), even without any cytopathic effect. While qPCR CT values/genome copy numbers can provide information about the amount of viral genetic material in a sample, they are not directly used to validate virus inactivation (Snipaitiene et al., 2023). This study followed a strategy to validate virus inactivation by comparing the amount of viral genetic material present at the beginning and after a certain period of cellular infection by qPCR. If the inactivation process is effective, there should not be any increase in genome copy numbers over the incubation period. In this study, heat inactivation of SARS-CoV-2 causes a decrease in genome copy numbers (**Figures 2C, D**, **3B, C**) or remains undetectable in RT-qPCR (**Figures 4B**, **5D**) over the incubation period, validating virus inactivation. A decrease in genome copy number is expected in the treated group due to RNA degradation at 37°C over time (Takeuchi et al., 2022) as reflected in **Figures 2D**, **3B**. To the contrary, the untreated virus should show a decrease in CT value or an increase in genome copy numbers over time in cell cultures, as demonstrated in this study (**Figures 2**–**5**). In this study, we followed two different experimental approaches for viral infection. In one approach, the virus (either treated or untreated) was incubated with the target cells for the entire duration of 72 hours, The virus genetic material was detected from the very beginning of the incubation period (**Figures 2D**, **3B**). In the second approach, cells were incubated with the virus just for one hour. After the initial one-hour incubation, the cells were washed to remove any remaining uninfected virus genetic materials, and fresh media was added to allow the cells to continue growing for 72 hours. The treated virus, which is expected to be non-infectious, failed to infect the cells during the first hour of incubation. As a result, it was undetectable in the samples taken after washing and remained undetectable throughout the 72-hour incubation period (**Figures 4B**, **5C, D**), validating inactivation.

For successful downstream applications, a quality check of post-inactivated samples is necessary. The choice of inactivation method may also vary based on desired downstream applications. If an extreme condition like a very high temperature alters an assay sensitivity, a lower temperature or another type of inactivation method can be adopted without altering the validation procedure. Lateral-flow immunoassay-based SARS-CoV-2 antigen tests are a quick alternative to the RT‐PCR method. While the tests are performed using untreated nasal or oral swabs diluted in a solution provided in the kit, handling positive samples or discarding the used kit with positive samples is not safe and can cause the spread of disease. We performed lateral-flow immunoassay using an over-the-counter COVID-19 Ag test kit using various heat-treated SARS-CoV-2 positive samples (pure virus culture, saliva, and nasal swab samples) to check the antigen quality of the post-treated samples. Based on the viral load, faint or strong bands were noticed in the COVID-19 Ag tests (**Figure 6A**). Generally, lateral flow rapid antigen test kits exhibit lower sensitivity compared to molecular tests such as qPCR. These kits may fail to detect samples with very low viral titers or with major mutational changes (**Supplementary Figure S2**). Even though the sensitivity of RT-qPCR assays for detecting SARS-CoV-2 in samples may be reduced following treatment of the samples at higher temperatures (Burton et al., 2021), all heat-treated samples tested positive in RT-qPCR in this study (**Figure 6B**). We also successfully performed whole genome sequencing of SARS-CoV-2 isolated from heat-treated saliva samples (**Table 1**).

**Figure 7:**
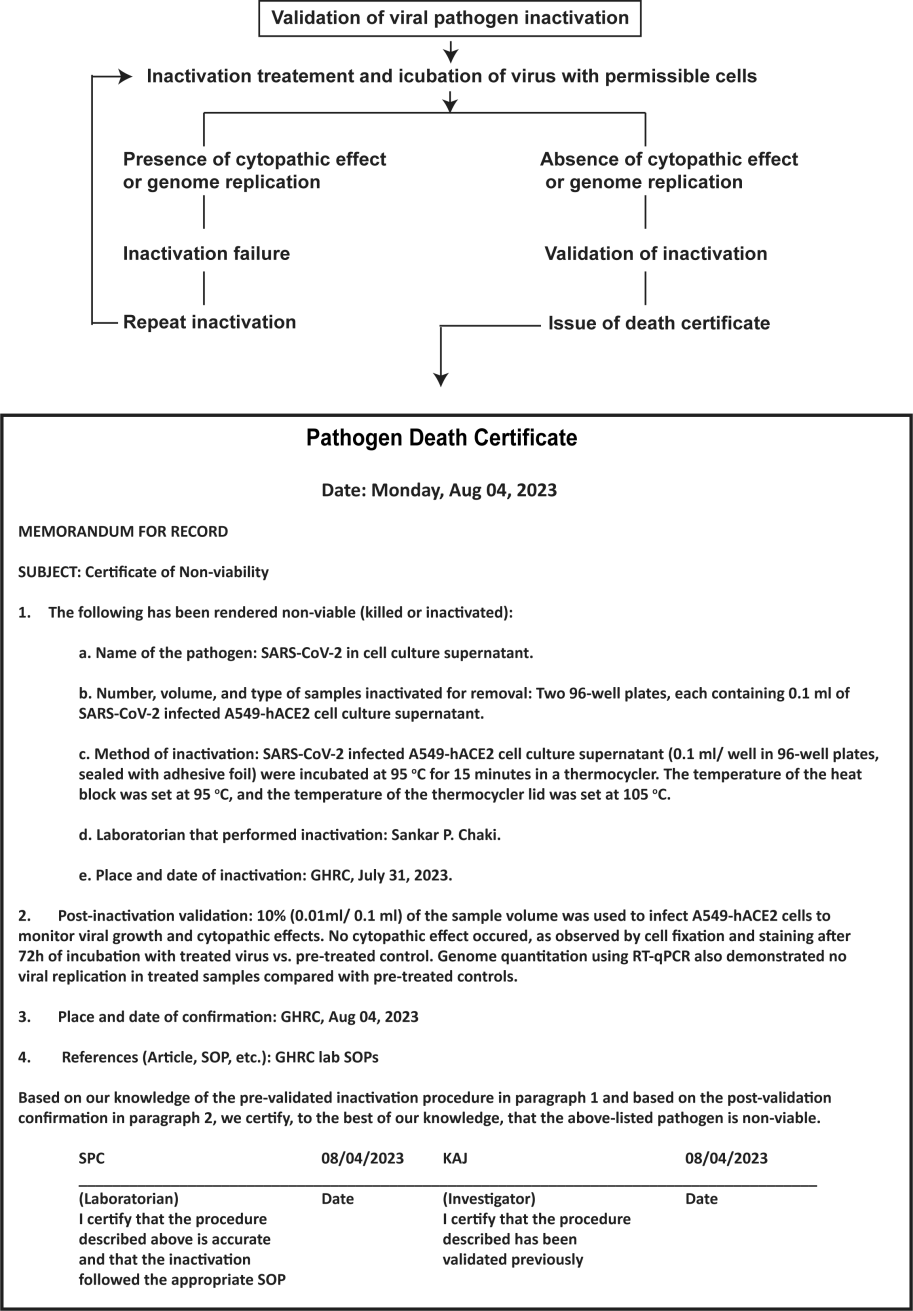
Validation steps and documentation of pathogen inactivation. This illustration shows a simplified flowchart of the steps to validate pathogen inactivation. It includes a pathogen death certificate with information on the pathogen, inactivation location and date, inactivation method, post-inactivation validation, and signatures of lab personnel and the project investigator. This type of inactivation certification process is useful for documenting the transfer of inactivated materials from high-containment labs and may also be archived for institutional record-keeping.

In this study, we used SARS-CoV-2, a BSL3 agent but not a select agent, to demonstrate validation of pathogen inactivation. BSL-3 laboratories handle a wide range of bacterial or viral pathogens, including *Mycobacterium tuberculosis*, *Brucella* species, yellow fever virus, various species of *Rickettsia*, West Nile Virus, certain strains of avian influenza such as H5N1 and MERS-CoV. Although our validation strategy in this study is focused on SARS-CoV-2 viral pathogens, it can be extended to other pathogen types with some modifications. qPCR strategy might be adopted for any pathogen type with a known genome sequence. The federal select agent program requires an inactivation or death certificate after viability assessment for removing select agents from a BSL3 or BSL4 laboratory. A copy of an inactivation certificate must accompany the inactivated material when the inactivated material is transferred externally or internally and maintain the record for at least three years (HHS, 2020). Although no specific prescribed format is required, an example of an inactivation or death certificate is shown in this study (**Figure 7**). Failing to comply with biosafety protocols increases the likelihood of recontamination, especially during the interim storage phase when validation procedures are underway and in the course of sample removal. An express statement should be included to ensure that the work conducted by the receiving laboratory using this material is executed in a safe and contained manner minimizing any potential risk to their laboratory scientists. Additionally, suppose the receiving laboratory observes any growth in the inactivated samples. In that case, it is the responsibility of the receiving party to notify the sender and other appropriate parties immediately and secure the sample.

## 5 Limitation and recommendation

High temperatures can lead to RNA degradation in pathogen inactivation processes. Viral pathogens can be inactivated using various methods of choice before applying this validation strategy. However, this validation method relies on cell culture systems, which require specific cell types to support viral replication and detect cytopathic effects. To overcome limitations with viruses that are challenging to grow in standard laboratory cell lines, researchers can explore alternative approaches. These may involve using primary cells from the host, co-culturing cells, or genetically modifying cells to express specific receptors. Before applying this method in high-containment laboratories, it’s crucial to conduct revalidation using different controls, including live and inactivated pathogens. A 72-hour incubation period is preferable to allow enough time for viral infection and growth while monitoring for cytopathic effects or changes in genome replication. RNA degradation in inactivated samples over 72 hours leads to improved differentiation in genome replication compared to untreated control samples, aiding the validation process.

## 6 Conclusion

This study introduced a simultaneous dual validation strategy for ensuring the inactivation of viral pathogens in high-containment laboratory. The study used SARS-CoV-2 wild type, delta, and omicron variants and two cell culture models: A549-hACE2 and Vero E6-TMPRSS2-T2A-ACE2. The approach involved two key elements: 1) qualitative assessment of cytopathic effects and 2) molecular quantitation of viral genome replication through RT-qPCR, both carried out simultaneously. Post-heat-inactivated samples retained characteristics suitable for molecular testing, as evidenced by successful COVID-19 antigen testing, RT-qPCR, and whole genome sequencing. Although inactivation method can vary based on downstream applications, this dual validation strategy can be adopted to ensure inactivation success while working with different viral pathogens in high-containment laboratories. Its main purpose is to expedite and streamline clinical diagnosis and biomedical research while improving the biosafety and biosecurity of sample transfer from high-containment laboratories. In addition, this validation strategy can be applied in various drug safety and efficacy studies in cell culture models.

## Data availability statement

The datasets presented in this study can be found in online repositories. The names of the repository/repositories and accession number(s) can be found below: https://gisaid.org/, EPI_ISL_1626969, EPI_ISL_2365356, EPI_ISL_2340581, EPI_ISL_2340662, EPI_ISL_2340614, EPI_ISL_2340615, EPI_ISL_2340626, EPI_ISL_2340570, EPI_ISL_2340575, EPI_ISL_2402571, EPI_ISL_13295050, EPI_ISL_10271777, EPI_ISL_17371329.

## Ethics statement

De-identified saliva or nasal swab samples in viral transport media (VTM) were received from the COVID-19 surveillance screening program. The Texas A&M University Institutional Review Board determined this study was not human subject research. Virus isolation from nasal swab samples was determined as non-human research (IRB2023-0941).

## Author contributions

SC: Conceptualization, Data curation, Formal Analysis, Investigation, Methodology, Software, Supervision, Validation, Visualization, Writing – original draft, Writing – review & editing. MK-M: Investigation, Resources, Visualization, Writing – review & editing. BN: Formal Analysis, Methodology, Writing – review & editing. KZ: Investigation, Project administration, Resources, Writing – review & editing.
