# Supplementary material for: Validating the inactivation of viral pathogens with a focus on SARS-CoV-2 to safely transfer samples from high-containment laboratories
Source: Front Cell Infect Microbiol. 2024 Mar 6;14:1292467. doi: 10.3389/fcimb.2024.1292467 (PMC10951993; doi:10.3389/fcimb.2024.1292467)
Supplement: Supplementary file 1 [file DataSheet_1.docx]

Supplementary Material

# Supplementary Figures


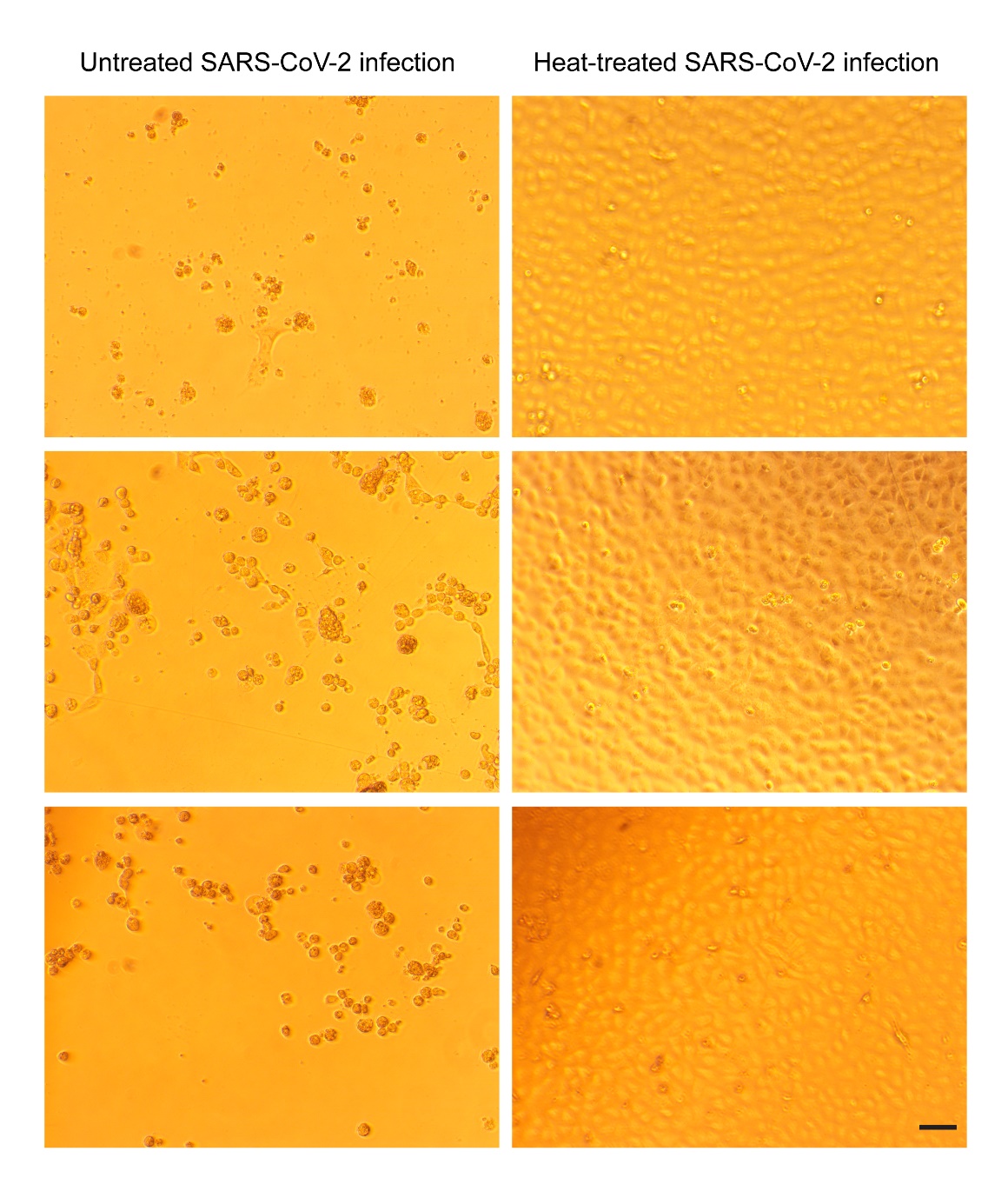


**Figure S1.** Cytopathic effect in A549-hACE2 cells after 72h of viral infection. The presence or absence of cytopathic effect was observed in A549-hACE2 cells under a bright-field microscope following 72h of incubation with untreated or heat-treated (95 ^o^C for 10 min) SARS-CoV-2 delta variant. The scale bar represents 50 micrometers.


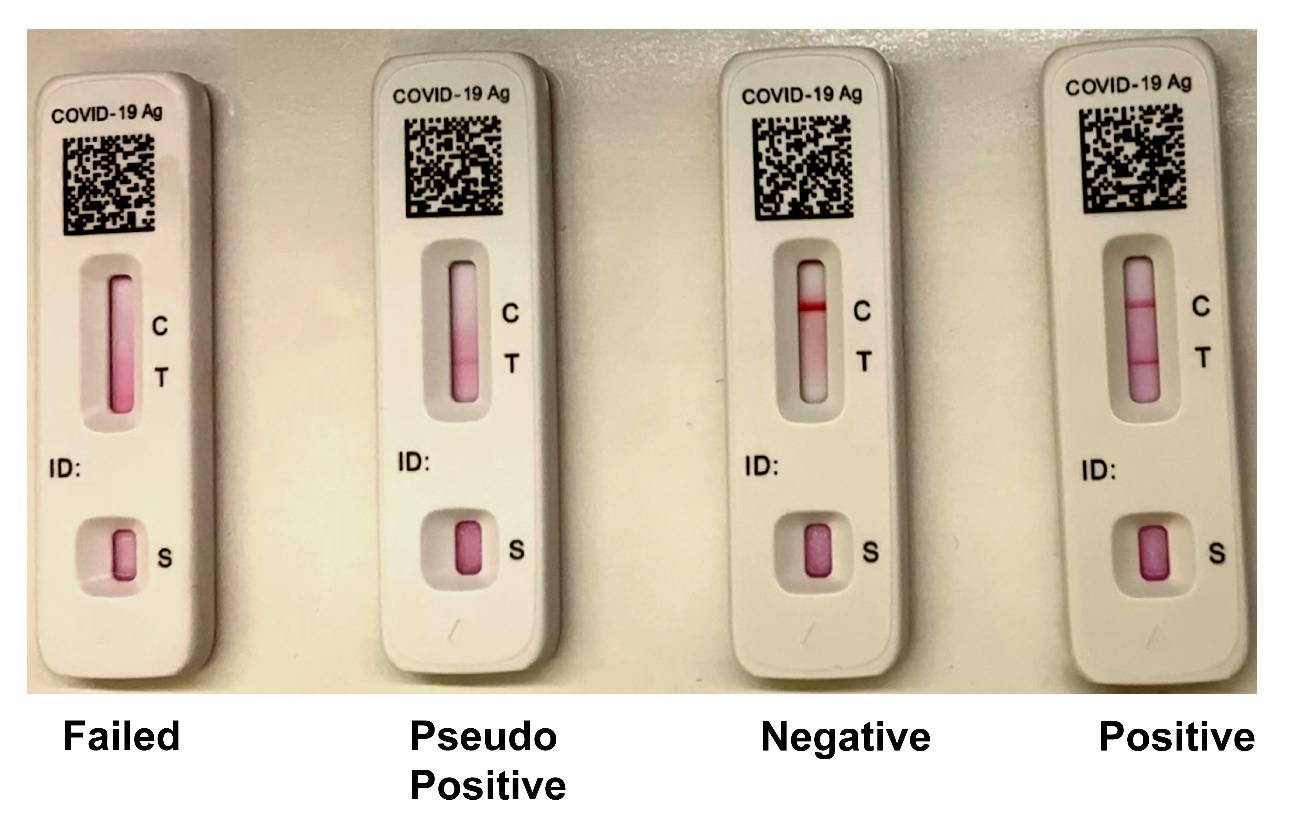


**Figure S2.** **Inconclusive COVID-19 antigen testing with heat-inactivated nasal swabs.** Representative images of COVID-19 antigen testing using heat-treated nasal swab samples demonstrate inconclusive outcomes due to the absence of control bands in the initial two samples. Although a test band is visible in the second sample, its lack of a control band resulted in its classification as pseudo-positive.
